# Surprising Visitor at Emergency Operating Table: *Taenia saginata*

**DOI:** 10.4274/balkanmedj.galenos.2019.2019.11.67

**Published:** 2020-04-10

**Authors:** Nermin Şakru, Serhat Oğuz, Cemal Çiçek, Hüseyin Aksoy, Mehmet Ali Yağcı

**Affiliations:** 1 Department of Medical Microbiology, Trakya University School of Medicine, Edirne, Turkey; 2 Department of General Surgery, Trakya University School of Medicine, Edirne, Turkey; 3 Department of Medical Microbiology, Aksaray Training and Research Hospital, Aksaray, Turkey; 4 Clinic of General Surgery, Sorgun Güven Hospital, Yozgat, Turkey; 5 Clinic of General Surgery, Anka Hospital, Gaziantep, Turkey

Only kings, presidents, editors, and people with tapeworms have the right to use the editorial “we.” Mark Twain (1).

A 53-year-old woman with a history of in-vehicle injury was admitted to the emergency department of Trakya University Hospital. Initial examination revealed that the arterial blood pressure, pulse, and respiratory rate were 90/60 mmHg, 104/min, and 22/min, respectively. Diffuse tenderness was observed during the abdominal examination; however, no defensive and rebound findings were found. The laboratory parameters did not show any specific values. Abdominal ultrasonography exhibited intraperitoneal free fluid, and computed tomography showed active intraperitoneal hemorrhage along with hematoma in the mesenteric area. The symptoms of peritoneal irritation in the upper abdominal quadrant along with imaging studies suggested a surgically acute abdomen. Afterward, urgent exploration by laparotomy was performed. Multiple injuries and bleeding foci were detected in the small intestine meso during the surgical exploration. The circulation was disrupted with no peristalsis in the small intestine segment of about 80 cm at a distance of 180 cm from the ligament of Treitz. Furthermore, segmental small bowel resection was performed. During anastomosis, a tapeworm was observed to be moving from the intestine into the abdominal cavity. The tapeworm, almost 4 m long and encountered by chance, was carefully removed from the abdominal area and sent to the laboratory (Figure 1). After the laboratory examination, we confirmed that it was *Taenia saginata (T. saginata)*. It was motile and the scolex had 4 suckers but lacked the rostellum and rostellar hooks (Figure 2). The number of proglottids was nearly 1000, and the premature proglottids were wider than the length. It survived in the laboratory for a week. Live images were obtained during this time (Supplementary Video). No drugs were administered to the patient for the parasite.

*T. saginata* is distributed among humans globally, including Turkey, through various sources. Infection is due to nutritional habits, such as eating raw or undercooked beef as observed in our case. The cases with T. saginata are usually asymptomatic. The scolex of *T. saginata* has 4 suckers without hooklets, whereas the scolex of *T. solium* is armed with hooklets (1,2). In literature, the imaging methods, such as conventional endoscopy, colonoscopy, or capsule endoscopy, have shown the in vivo imaging of tapeworms in few cases; however, it is difficult to remove the scolex of tapeworms despite administration of oral gastrografin or praziquantel (3,4).

In this case, we completely removed the living *T. saginata* with scolex at the operating table. The scolex and premature proglottids of live *T. saginata* were observed in detail using microscopy.

## Figures and Tables

**Figure 1:**
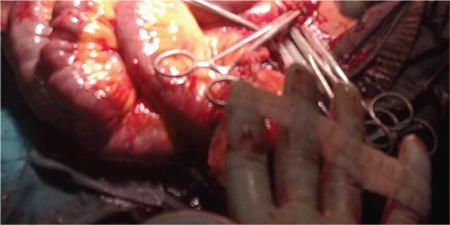
The parasite (on the fingers) was carefully removed from the abdomen during the surgery.

**Figure 2:**
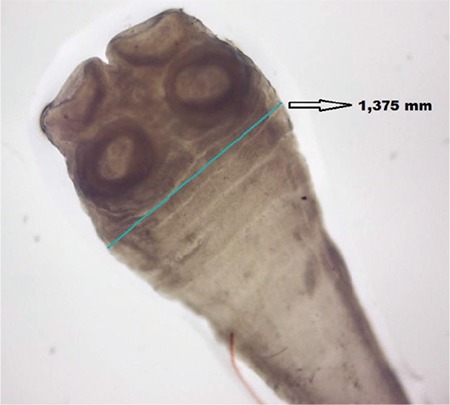
The scolex of *Taenia saginata* is nearly 1,4 mm (X40).
